# Diagnostic performance of reticulocyte hemoglobin equivalent in assessing the iron status

**DOI:** 10.1002/jcla.23225

**Published:** 2020-02-11

**Authors:** Pawadee Chinudomwong, Aleeyas Binyasing, Rangsiri Trongsakul, Karan Paisooksantivatana

**Affiliations:** ^1^ Department of Pathology Faculty of Medicine Ramathibodi Hospital Mahidol University Bangkok Thailand

**Keywords:** diagnostic performance, exclusion, inflammation, iron deficiency anemia, reticulocyte hemoglobin equivalent (RET‐He)

## Abstract

**Background:**

Measurement of reticulocyte hemoglobin equivalent (RET‐He) is rapid, convenient, and cost‐effective. Yet, researches on its performance in diagnosing iron deficiency with concurrent inflammation are limited. Hence, this study investigated RET‐He value in various states, including inflammation, and evaluated its diagnostic performance in iron status assessment.

**Methods:**

Retrospectively, 953 clinical data and laboratory results—complete blood count, reticulocyte count, RET‐He, and serum ferritin—were reviewed. Patients on iron therapy were excluded. Iron status was defined by serum ferritin as the reference method. RET‐He among populations was investigated. Its diagnostic performance and optimal cutoff were determined by ROC analysis.

**Results:**

Three population groups were classified: healthy control, iron deficiency anemia (IDA), and non‐ID anemia. Significantly, RET‐He value in IDA was lower than that of healthy control, anemia of inflammation, and chronic kidney disease (*P* < .0001). Low RET‐He was also observed in IDA with concomitant inflammation despite normal‐to‐high serum ferritin levels. No significant difference was observed between RET‐He values in pure IDA and thalassemia (*P* = .57). ROC curve analysis revealed AUC of 0.876 (*P* < .0001) at cutoff 30 pg, by which IDA was discriminated with 74.2% sensitivity and 97.4% specificity. Applying cutoff ≤30 pg, IDA can be diagnosed with 96% sensitivity, 97.4% specificity, 80% PPV, and 99.6% NPV. Hence, RET‐He >30 pg signifies a non‐IDA state.

**Conclusion:**

In addition to convenience and cost‐effectiveness, RET‐He cutoff >30 pg can be potentially used to exclude IDA due to its excellent diagnostic sensitivity and specificity.

## 1 INTRODUCTION

Iron deficiency (ID) is well recognized as the most common nutritional deficiency^1^, ^2^ disorder in the world^3^ and the principal cause of global anemia,^4^ leading to adverse sequelae, namely, growth retardation,^5^, ^6^ neurocognitive deficit,^1^ impaired immune system,^3^ and increased risk of prematurity and maternal mortality.^7^ Early detection is, thus, crucial for proper and timely management to prevent such consequences. Presently, there is still no single best test for diagnosing this condition. Bone marrow iron staining, the gold standard method for diagnosing iron deficiency, is invasive^8^, ^9^, ^10^, ^11^ and subjective.^9^, ^10^ Conventional iron biomarkers are also affected by acute‐phase responses,^8^, ^12^ and influenced by diurnal variation and dietary intake.^12^, ^13^

Due to these diagnostic difficulties, an alternative marker is sought. Reticulocyte hemoglobin equivalent (RET‐He) is one of potential markers. Its measurement is not only rapid but also convenient and cost‐effective. Introduced in 2005, studies evaluating RET‐He for its performance in diagnosing iron deficiency and application in various clinical settings are numerous.^13^, ^14^, ^15^, ^16^, ^17^, ^18^, ^19^ However, investigations on RET‐He in the diagnosis of ID with coexistence of inflammatory conditions are limited.^4^, ^20^, ^21^ This study, thus, aimed to investigate RET‐He value in various conditions, including inflammation‐related states, and evaluate its diagnostic performance in assessing the iron status. A diagnostic algorithm was, then, proposed.

## 2 METHODS

### 2.1 Study design

This was a retrospective analytical study, performed at Ramathibodi Hospital, Bangkok. An ethical approval was obtained from the Ethics Committee for Human Research of Ramathibodi Hospital, Mahidol University (MURA 2017/509). Nine hundred and fifty‐three clinical data—collected from April 2017 to May 2018—were reviewed in conjunction with laboratory results, that is, complete blood count (CBC), reticulocyte count, RET‐He, and serum ferritin. Patients on iron therapy (n = 15) were excluded.

### 2.2 Definition of iron status and anemia

Iron status was defined by serum ferritin as the reference method. Low serum ferritin (<33.71 pmol/L or 15 ng/mL according to the World Health Organization [WHO]; 1 ng/mL = 2.247 pmol/L) signified ID in both women and men.^22^ Anemia was defined, following the WHO criteria, by hemoglobin (Hb) level <120 g/L in women (W) or <130 g/L in men (M). Iron deficiency anemia (IDA) was ID‐associated anemia. IDA with concomitant inflammation (IDA‐inf) and anemia of inflammation (AI) were similar in terms of anemia with normal or high serum ferritin level (≥33.71 pmol/L), and a history of associated inflammatory conditions, namely, chronic infections or non‐infectious inflammation, and malignant diseases. Differences between the two were the characteristic of anemia: microcytic hypochromic in IDA‐inf versus normocytic normochromic anemia in AI. Thalassemia was defined by Hb typing and/or DNA analysis results, demonstrating alpha and/or beta thalassemia.

### 2.3 Study populations

**Figure 1:**
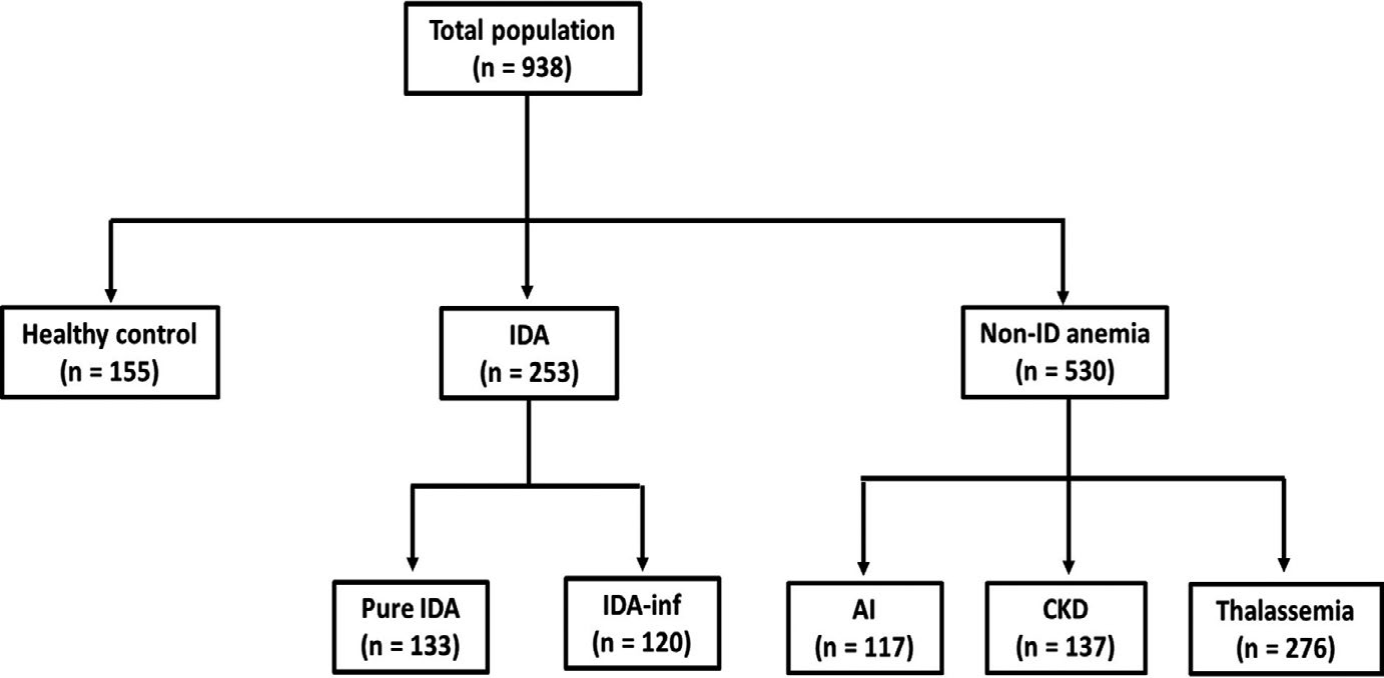
Study populations. Diagnostic criteria for each population group: (1) Healthy control: absence of clinical symptom, Hb ≥ 120 g/L (W) or ≥ 130 g/L (M), MCV 80‐100 fL, MCH 27‐31 pg, WBC count 4.0‐10.0 x 10^9^/L, platelet count 150‐450 x 10^9^/L, and serum ferritin 33.71‐674.16 pmol/L; (2) IDA: Hb < 120 g/L (W) or < 130 g/L (M), MCV < 80 fL, MCH < 27 pg, Reticulocyte Production Index (RPI) <2, and serum ferritin < 33.71 pmol/L or ferritin ≥ 33.71 pmol/L with clinical diagnosis of IDA; (3) IDA‐inf: Hb < 120 g/L (W) or < 130 g/L (M), MCV < 80 fL, MCH < 27 pg, and serum ferritin ≥ 33.71 pmol/L in presence of inflammation; (4) Anemia of inflammation: Hb < 120 g/L (W) or < 130 g/L (M), MCV 80‐100 fL, MCH 27‐31 pg, and serum ferritin ≥ 33.71 pmol/L with associated inflammatory conditions; (5) Anemia of chronic renal insufficiency: Hb < 120 g/L (W) or < 130 g/L (M), MCV 80‐100 fL, MCH 27‐31 pg, and serum ferritin ≥ 33.71 pmol/L in patients diagnosed of CKD; (6) Thalassemia: Hb < 120 g/L (W) or < 130 g/L (M), MCV < 80 fL, MCH <27 pg, and Hb typing and/or DNA analysis results demonstrating alpha and/or beta thalassemia

By the above criteria, three population groups were classified, for instance, healthy control (n = 155), IDA (n = 253) including pure IDA (n = 133) and IDA‐inf (n = 120), and non‐ID anemia group comprising AI (n = 117), chronic kidney disease (CKD) (n = 137), and thalassemia (n = 276) (Figure 1).

### 2.4 Laboratory methods

CBC and reticulocyte analysis were performed on Sysmex XN‐10 hematology analyzer (Sysmex Corporation). Serum ferritin was quantified by using ARCHITECT ferritin assay, a chemiluminescent microparticle immunoassay (Abbott Laboratories). Determination of reticulocyte hemoglobin content is conducted in the reticulocyte channel, composing of two processes. Firstly, nucleic acid is stained^23^ with polymethine dye,^19^, ^24^ which is specific for RNA/DNA.^24^ Then, measurement of reticulocyte cellular hemoglobin was done based on fluorescent flow cytometry technique.^19^, ^23^, ^24^

The 2D‐scattergram depicts the measurement of mature erythrocytes and reticulocytes. On the *y*‐axis, the forward light scatter demonstrates an individual cell size, plotted against fluorescence intensity—a measure of RNA content—on the *x*‐axis. The average value of forward‐scattered light intensity of reticulocytes is expressed as a log transformation of Ret‐Y.^23^ The results are presented as picograms (pg) of Hb per reticulocyte.^8^ This obtained reticulocyte hemoglobin content is termed RET‐He parameter.

### 2.5 Precision studies

Precision analysis for hematology analyzer was carried out, following the International Council for Standardization in Haematology (ICSH) guidelines,^25^ prior to sample testing. Repeatability (within‐run precision) test was performed by a single run of 20 measurements^26^ on each of the three levels (low, normal, and high) of quality control materials (XN CHECK). Between‐run precision test was carried out by running a single measurement on the above control materials daily over a period of 20 days.^25^, ^26^

### 2.6 Statistical analysis

Data analysis was carried out using MedCalc statistical software version 18 (MedCalc Software). Data normality was assessed by Kolmogorov‐Smirnov test. Non‐parametric data were described as median and interquartile range (IQR). Comparison of data between two groups employed Mann‐Whitney *U* test and Kruskal‐Wallis test for multiple groups. *P* value <.05 indicated statistical significance. ROC analysis was performed to evaluate the diagnostic performance of RET‐He and determine its optimal cutoff value in distinguishing IDA.

## 3 RESULTS

### 3.1 Precision studies

Precision analysis for RET‐He revealed a low imprecision for the hematology analyzer employed in this study. A standard deviation of <0.5 pg with %CV of <2% in every level of the tested control materials was observed, falling within 5% limit specified by the manufacturer.

### 3.2 Demographic data of the study populations

Table 1 illustrates the demographic data and laboratory parameters of the study populations. Overall, most of the healthy control and patients in every group were women. Evidently, the prevalence of IDA was much higher in women than in men, ranging from two folds (80 women vs 40 men) in IDA‐inf to four folds (108 women vs 25 men) in pure IDA. Also, IDA was observed most in women of child‐bearing age (median of 34 years in pure IDA and 45 years in IDA‐inf) while inflammation‐related conditions like AI and CKD were observed more among the elderly.

**Table 1:** Data of study populations

Items	Healthy control	IDA		Non‐ID anemia		
		pure IDA	IDA‐inf	AI	CKD	Thalassemia
	(n = 155)	(n = 133)	(n = 120)	(n = 177)	(n = 137)	(n = 276)
Age (y)	15	34	45	73	70	41
Sex						
Female	89	108	80	70	76	180
Male	66	25	40	47	61	96
Laboratory parameters						
Hb (g/L)	139 (18)	93 (35)	103 (18)	98 (23)	96 (19)	89 (28)
MCV (fL)	86.1 (4.25)	66.2 (14.60)	69.2 (11.40)	89.0 (5.70)	88.8 (5.60)	64.5 (15.08)
MCH (pg)	28.7 (1.30)	19.8 (6.40)	21.8 (4.45)	29.1 (2.10)	29.0 (2.20)	20.3 (4.55)
RET‐He (pg)	33.0 (1.45)	20.6 (9.00)	25.2 (6.15)	32.2 (3.9)	31.8 (3.53)	19.6 (5.33)
Ferritin (pmol/L)	119.54 (102.80)	13.03 (10.79)	125 (281.89)	919.02 (2085.22)	806.11 (1205.18)	740.16 (1626.27)

Note

Demographic data and laboratory parameters of the study population. Data are expressed in median (IQR).

When IDA occurred concurrently with inflammation, a low RET‐He value (median 25.2 pg, IQR 6.15) was detected despite raised serum ferritin levels (median 125 pmol/L, IQR 281.89), implying that RET‐He was not affected by inflammation. The finding further showed that although hematologic parameters in pure IDA and thalassemia were similar, serum ferritin levels between the two differed significantly (median 13.03 vs 740.16 pmol/L; *P* < .0001).

### 3.3 RET‐He values among the population groups

**Figure 2:**
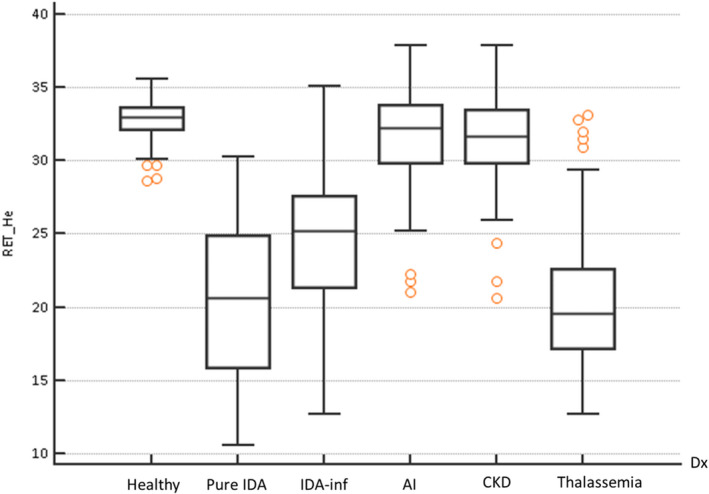
RET‐He values among the population groups. Box‐and‐Whisker plot illustrates RET‐He values among six population groups in this study. Comparison of values between groups is performed by the Kruskal‐Wallis test (*P* < .05)

Comparison analysis revealed that RET‐He in healthy control differed significantly from that of the patient group (median 33.0 pg vs 24.8 pg; *P* < .0001). Significantly, RET‐He in IDA (median 22.5 pg, IQR 7.3) was lower than that of healthy control (median 33.0 pg, IQR 1.45; *P* < .0001), and non‐ID group including AI (median 32.2 pg, IQR 3.90; *P* < .0001), and CKD (median 31.7 pg, IQR 3.53; *P* < .0001). No significant difference was observed between RET‐He values in pure IDA and thalassemia (median 20.6 pg, IQR 9.0 and 19.6 pg, IQR 5.33, respectively; *P* = .57) as well as between AI and CKD (median 32.2 vs 31.7 pg; *P* = .38). Among the inflammatory conditions, IDA‐inf demonstrated the lowest RET‐He value as compared to AI and CKD (Figure 2).

### 3.4 Optimal RET‐He cutoff for IDA detection

**Figure 3:**
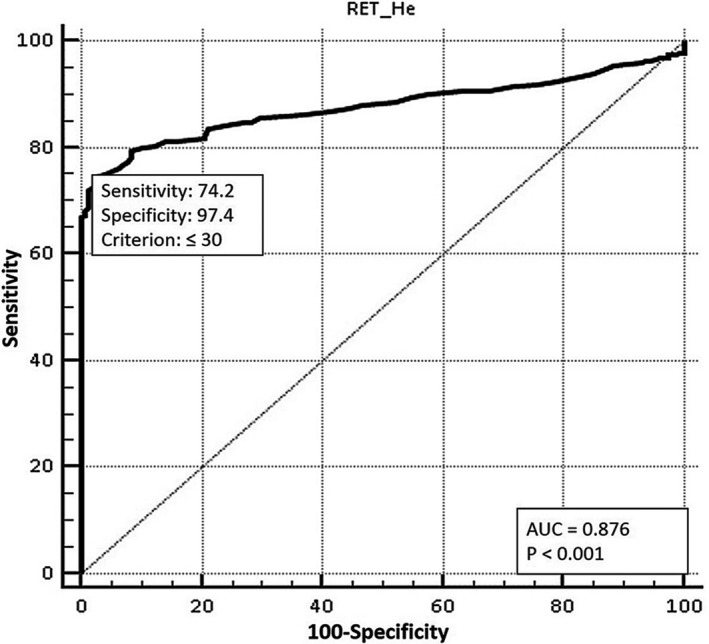
Optimal RET‐He cutoff. RET‐He cutoff value in distinguishing IDA is optimally determined by ROC analysis. Cutoff ≤30 pg demonstrates AUC of 0.876 (74.2% sensitivity, 97.4% specificity; *P* < .001)

By ROC analysis, the optimal cutoff for RET‐He in IDA detection was generated from the best combination of sensitivity and specificity. ROC curve revealed area under the curve of 0.876 (95% CI 0.854‐0.897; *P* < .001) at cutoff ≤30 pg (Figure 3), at which IDA was distinguished with 74.2% sensitivity and 97.4% specificity. Applying this cutoff, IDA could be diagnosed with 96% sensitivity, 97.4% specificity, 80% positive predictive value (PPV), 99.6% negative predictive value (NPV), and positive likelihood ratio (LR+) of 37.2 (95% CI 14.1‐97.9). RET‐He >30 pg, hence, implied the non‐IDA state.

## 4 DISCUSSION

Reticulocyte hemoglobin content has been reported as a marker that provides a snapshot of iron availability for erythropoiesis^13^, ^23^, ^27^, ^28^ in the bone marrow,^13^, ^27^ an indicator of iron therapy response,^28^, ^29^, ^30^ and an early marker of iron‐deficient erythropoiesis.^20^, ^29^, ^31^ It enables an early detection^23^ and indication^29^ of ID owing to not only its short life span (1‐2 days^8^, ^23^, ^29^, ^30^) in the circulation as compared to mature erythrocytes^23^ (120 days^32^) but also its better predictive value^28^ than hemocytometric parameters, for example, MCV, red cell distribution width (RDW), and Hb.^17^ These are insensitive markers in detecting early erythropoietic disturbances^33^ as their changes (MCV: 21 days, RDW: 30 days, and Hb: 60 days^17^) are observed when IDA has already taken place. In contrast, RET‐He is the first peripheral blood count marker being abnormal in the presence of iron deficiency.^34^ It is also an early indicator of response to iron therapy, of which effect is detected about two days after initiation of the optimal treatment^31^ as opposed to ferritin, of which first response occurs in 1‐2 weeks.^34^

Since its introduction by Sysmex in 2005,^35^ several studies have been carried out to investigate the clinical application of RET‐He. The reported findings are in consistence with one another in that RET‐He is a helpful tool in diagnosing iron‐restricted erythropoiesis,^24^ ID,^8^, ^14^, ^35^, ^36^ and IDA.^9^, ^16^, ^32^ Despite of that, only few researches involve ID with concomitant inflammatory states in the study group.^4^, ^20^, ^21^ This study, therefore, aims to extensively investigate RET‐He values in a variety of conditions, involving inflammation, and evaluate its diagnostic performance in assessing the iron status.

Clinical data and laboratory results are integrated for the analysis. Apparently, the finding that IDA possesses low RET‐He is consistent with previous studies, supporting its clinical usefulness reported earlier. In this research, at RET‐He cutoff >30 pg, IDA can be excellently ruled out. In contrast, at RET‐He ≤30 pg, the finding is slightly complicated. Because, in the real world, IDA can occur not only singly but also commonly with several non‐ID conditions, RET‐He value ≤30 pg cannot evidently distinguish IDA from these situations. As such, a diagnostic algorithm is proposed.

RET‐He is incorporated as the initial test in the algorithm because of not only its outstanding diagnostic performance but also several analytical advantages. That is, its determination is fully automated^14^ with only 2‐step process and can be performed on the same specimen as for CBC.^23^ This renders simplicity and convenience,^17^ rapidity^14^, ^17^ with measurement in <2 min,^14^ and cost‐effectiveness.^23^ The result is, then, readily reported as a part of CBC. Aside from up‐to‐3‐days stability, RET‐He measurement demonstrates low total, analytical, and biological variations (median 2.1%, 1.6%, and 1.7%, respectively).^37^ The precision studies performed in this research also show good reproducibility and low imprecision in RET‐He determination.

**Figure 4:**
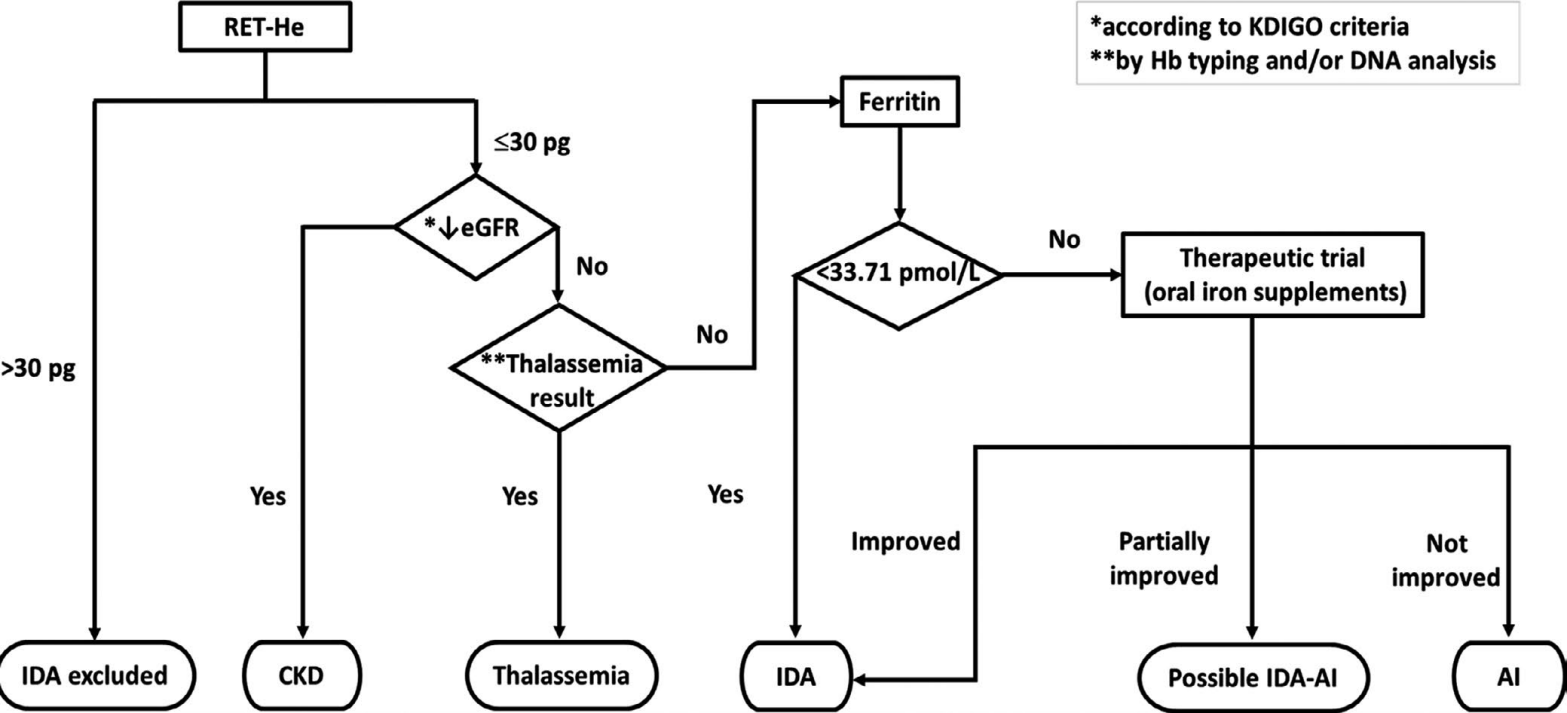
Proposed diagnostic algorithm. Evaluation of RET‐He is incorporated into the proposed diagnostic algorithm. At RET‐He cutoff >30 pg, IDA is excluded. As IDA can co‐exist with non‐ID conditions, ferritin still plays a role in the confirmation of such condition

Consistent with the proposed diagnostic algorithm (Figure 4), RET‐He >30 pg positively confirms the non‐IDA state. This facilitates the exclusion of IDA without necessity for further costly and time‐consuming iron study. Among the RET‐He ≤30 pg group, where IDA co‐exists with other non‐ID conditions, diagnosing a particular condition requires a specific investigation or criterion accordingly. In this sense, evaluation of serum ferritin still plays a role in the diagnosis of IDA. Low serum ferritin confirms IDA while a normal or high level cannot clearly exclude the condition because of its nature as an acute‐phase reactant. In such situation, therapeutic trial by oral iron supplementation is combined to assist the diagnosis.^38^, ^39^

From the result of IDA‐inf group, Ret‐He could be helpful in identifying IDA among individuals suffering from inflammatory disorders where IDA co‐exists because it is not affected by inflammation. Lastly, further analysis reveals an added clinical value of RET‐He. By using the above cutoff, the likelihood ratio of iron depletion, defined as low serum ferritin in absence of clinical symptom and anemia, is about seven times (LR + 7.3; 95% CI 1.8‐29.6) that of healthy control, implying its capability of predicting iron‐depleted state in otherwise healthy person.

Despite the mentioned potentials, some disadvantages of RET‐He need to be stated. Initially, although RET‐He is hypothetically able to indicate an early response to iron therapy according to previous studies, the endpoint of treatment monitoring or follow‐up is the restoration of iron store, in which ferritin evaluation is indispensable. Next, though this parameter can be employed as a marker for predicting iron depletion in otherwise healthy population, confirmation essentially requires determination of serum ferritin. What is more, in countries where thalassemia is prevalent like Thailand, this parameter may not be suitable for assessing the iron status in the presence of alpha and beta thalassemia.^27^, ^40^ This is supported by a previous study, summarizing that sensitivity and specificity of reticulocyte hemoglobin content cutoff were insufficient to distinguish thalassemia or hemoglobinopathy carriers from IDA.y.^41^ As such, further studies are necessary.

To our knowledge, this is the first study that collects such a large sample size for the investigation. This is also one of a few researches that inflammation is considered for RET‐He assessment. Despite such strengths, limitations exist. Firstly, this study is conducted on Sysmex XN‐series hematology analyzer, which might not be available in many institutions, particularly those providing primary health care. This is probably the reason why the use of this parameter is still of limit despite numerous studies over years. Next, clinical diagnoses of the patient are extremely diverse, and more than one diagnoses are concurrently found in each patient, making the analysis difficult. Lastly, the current collection of data limits the utilization of RET‐He as a tool for iron therapy monitoring due to its retrospective nature. Yet, potential still exists for it to be employed as a follow‐up parameter. Further study is warranted to investigate and validate its feasible utility in this clinical setting.

## 5 CONCLUSION

In addition to its rapid, convenient, and cost‐effective measurement, RET‐He >30 pg is a potential marker in ruling out IDA with an excellent diagnostic sensitivity and specificity. However, when IDA co‐exists with other non‐ID conditions, which is common in actual clinical practice, evaluation of serum ferritin remains necessary for making the diagnosis.
